# A Method for Distinctly Marking Honey Bees, *Apis mellifera*, Originating from Multiple Apiary Locations

**DOI:** 10.1673/031.011.14301

**Published:** 2011-11-02

**Authors:** James Hagler, Shannon Mueller, Larry R. Teuber, Allen Van Deynze, Joe Martin

**Affiliations:** ^1^ Arid Land Agricultural Research Center, USDA-ARS, 21881 North Cardon Lane, Maricopa, AZ 85138 USA; ^2^ University of California Cooperative Extension, 1720 S. Maple Avenue, Fresno, CA 93702 USA; ^3^ University of California, Department of Plant Sciences, Mail Stop 1, One Shields Avenue, Davis, CA 95616 USA; ^4^ Carl Hayden Bee Research Center, USDA-ARS, 2000 E. Allen Road, Tucson, AZ 85719 USA (retired)

**Keywords:** ELISA, fluorescent powder, mark-capture, pollen mediated gene flow, protein marking, self-marking

## Abstract

Inexpensive and non-intrusive marking methods are essential to track natural behavior of insects for biological experiments. An inexpensive, easy to construct, and easy to install bee marking device is described in this paper. The device is mounted at the entrance of a standard honey bee *Apis mellifera* L. (Hymenoptera: Apidae) hive and is fitted with a removable tube that dispenses a powdered marker. Marking devices were installed on 80 honey bee colonies distributed in nine separate apiaries. Each device held a tube containing one of five colored fluorescent powders, or a combination of a fluorescent powder (either green or magenta) plus one of two protein powders, resulting in nine unique marks. The powdered protein markers included egg albumin from dry chicken egg whites and casein from dry powdered milk. The efficacy of the marking procedure for each of the unique markers was assessed on honey bees exiting each apiary. Each bee was examined, first by visual inspection for the presence of colored fluorescent powder and then by egg albumin and milk casein specific enzyme-linked immunosorbent assays (ELISA). Data indicated that all five of the colored fluorescent powders and both of the protein powders were effective honey bee markers. However, the fluorescent powders consistently yielded more reliable marks than the protein powders. In general, there was less than a 1% chance of obtaining a false positive colored or protein-marked bee, but the chance of obtaining a false negative marked bee was higher for “protein-marked” bees.

## Introduction

The honey bee self-marking method described here was developed specifically for use in a study to identify the dispersal patterns of bees throughout a 15.2 km^2^ commercial alfalfa seed production area containing genetically modified and non-genetically modified alfalfa fields. Our ultimate goal was to simultaneously mark as many honey bees as possible at each of nine different apiaries placed by the growers in the vicinity of these seed fields to serve as pollinators. It was imperative that the bees exiting each apiary simultaneously received a distinct mark so that the distance and direction traveled by marked bees collected in surrounding alfalfa fields could be precisely identified (Hagler et al, 2011).

In this paper, we describe the development of a bee marking device that attaches to the entrance of a commercial beehive. The device can be rapidly loaded with a portable dispenser tube containing a colored fluorescent powder or a combination of a colored powder and one of two protein-rich powders (i.e., a double mark). The protein powders tested included egg albumin from chicken egg whites and milk casein from cow's milk. The bees were self-marked with the various powders as they exited the hive through the device. The efficacy of the marking procedure was determined by first examining each bee for the presence of a fluorescent colored mark by direct visual inspection under magnification using ultraviolet light. Then each bee was analyzed for the presence of egg albumin protein and bovine casein protein using protein-specific enzyme-linked immunosorbent assays (ELISA) (Jones et al. 2006).

## Materials and Methods

### Bee marking device

**Figure 1.**
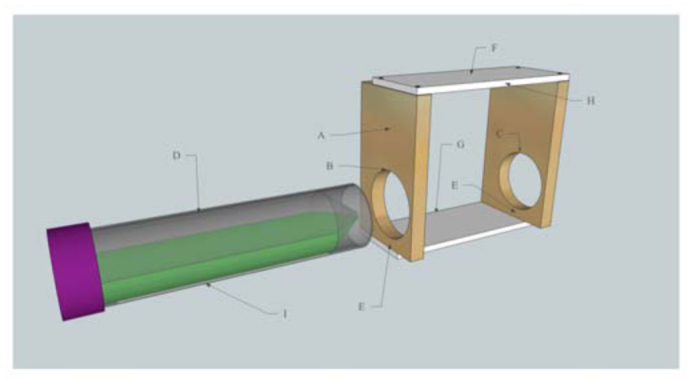
A diagram of the bee marking device and powdered marker dispenser tube. See ‘Materials and Methods’ for an explanation of the letter designations A through I. High quality figures are available online.

A diagram of the bee marking device is presented in Figure 1. The vertical edges of the device consist of two 73 × 44 × 7 mm wooden laths (Figure 1A). A 32 mm diameter hole is drilled into one vertical lath (Figure 1B), and a 30 mm diameter hole is drilled into the other lath (Figure 1C). These two holes hold a 50 mL plastic centrifuge tube (Figure 1D) that dispenses a powdered marker. The slight difference in the diameter of the two holes facilitates the insertion and removal of the dispenser tube from the apparatus. The bottom of each hole is precisely 5.0 mm from the bottom of each piece of vertical lath (Figure 1E). The top and bottom horizontal pieces of the device are 86 × 34 × 3 mm laths, nailed to the vertical laths using four small carpenter nails (Figure 1F). It is important that the bottom horizontal lath is flush with the front edge of the device (Figure 1G), and the top horizontal lath is flush with the back edge of the device (Figure 1H).

The bee marking device is glued to the hive entrance with latex caulking. The caulking is applied as a fine bead to the two vertical and top horizontal lath edges located on the backside of the device using a caulking gun. The device creates a 7.2 cm wide opening for the bees to enter and exit. Once the device is mounted onto a hive, the remaining length of the hive entrance is blocked with a nylon or metal screen to ensure that the bees can only enter or exit the hive through the marking device. The screen, while preventing entrance or exit, does not restrict air movement for hive ventilation.

### Powdered marker dispenser

The dispenser tube holding the various powdered markers is a 50 mL polypropylene centrifuge tube with a flat bottom (Figure 1D) (VWR International, www.vwrsp.com). An 85 × 12 mm rectangular opening is created on the wall of the tube using a cutting tool, such as a small utility knife. A fine bead of hot glue applied around the perimeter of the opening adheres a 110 × 30 mm piece of muslin fabric to the tube, covering the opening (Figure 1I). The muslin fabric is cut slightly larger than the hole in the tube so that it sags with the weight of the powdered marker, and provides a cushioned edge for the bees to brush up against as they exit the hive.

### Study site

**Figure 2.**
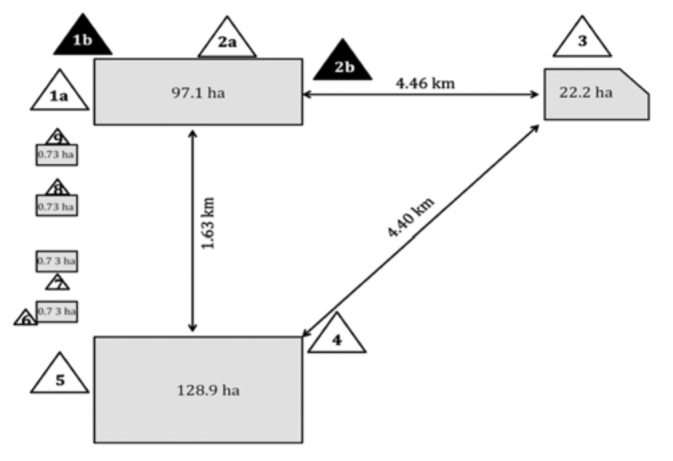
A diagram of the 15.2 km^2^ study area showing the location of each of the nine apiaries (number triangles) in relation to alfalfa seed fields (gray areas). Note that the apiaries designated as apiary 1 and 2 consisted of two nearby apiaries that were marked with green and blue fluorescent protein, respectively. Only bees collected in apiary 1a and 2a were used in this study. The size of each field in hectares (ha) is given in the shaded area. The 128.9 ha field contained herbicide-tolerant alfalfa (Roundup Ready), while the other fields contained conventional alfalfa that were susceptible to Roundup herbicide. High quality figures are available online.

The study area consisted of a 15.2 km^2^ agroecosystem dominated by alfalfa seed production fields located near San Joaquin, CA, USA. A schematic diagram of the study area depicting the location of the honey bee apiaries and blooming alfalfa fields is shown in Figure 2. The various apiaries were established near a 128.9 ha herbicide-tolerant (Roundup Ready, Monsanto Co., www.monsanto.com) alfalfa field and several conventional alfalfa seed fields that are susceptible to Roundup herbicide, ranging in size from 0.73 to 97.1 ha (Figure 2). At the onset of alfalfa bloom, hundreds of commercial honey bee colonies (3–4 story commercial beehives) were placed at the locations shown in Figure 2 at a density of 4.9 to 7.4 hives per hectare. This density of beehives provides alfalfa seed producers with the optimal density of pollinators needed to obtain maximum seed set (Mueller 2008). The number of marked hives and the specific marker(s) in each apiary is listed in Table 1. The large alfalfa fields had dozens of beehives placed at each apiary location, while the 0.73 ha fields had only four hives each. It was not feasible to install a marking device on every hive in the larger apiaries (e.g., apiaries 1–5, Figure 2). Therefore, only 9.1–13.3% of hives in these apiaries were fitted with a marking device (Table 1). Conversely, every hive (n = 4 per apiary) placed next to the small alfalfa fields was fitted with a marking device.

On 18 June 2007 a total of 80 marking devices were attached to the entrances of randomly selected hives in each apiary, now referred to as “marked hives” (Table 1). The remaining portion of each of these hive entrances was blocked with either nylon or wire window screen to facilitate airflow through the hive for thermoregulation. The bees were given approximately 44 hours to adjust to the alteration of the hive entrance. Then, on 20 June 2007, prior to the initiation of honey bee flight (e.g., before 07:00), a 50 mL dispenser tube (Figure 1D) containing one of five colored fluorescent powders or a colored powder (green or magenta) plus either powdered milk or egg white protein (mixed at a 1:1 ratio), was inserted into each marking device.

### Honey bee sampling procedures

Honey bees were collected at three locations within each apiary between 09:00 and 12:00 on 20 June 2007. Sampling locations included (1) entrances of unmarked hives, (2) entrances of marked hives, and (3) within the perimeter of each apiary. Individual honey bees were trapped in separate plastic bags as they exited marked and unmarked hives. Each sample bag was sealed and immediately frozen on dry ice. Approximately five bees were collected from four to six randomly chosen marked and unmarked hives within each large apiary (apiaries 1 through 5), and from all hives at apiaries 6 through 9 where every hive was fitted with a marking device. Free-flying bees were collected in the vicinity of each apiary by sweeping at chest height within the perimeter of each apiary for one min using a clean 38 cm diameter sweep net. The bees collected in the sweep nets were transferred into a plastic bag. The bag was sealed, rolled tightly to minimize bee movement within the bag, and immediately frozen on dry ice. All bee samples were placed into a -20° C freezer at the laboratory until analyzed for the presence of marks.

### Detection of fluorescent powders

Individual bees were removed from the freezer, placed under a 10× dissecting microscope (MEIJI Model EMZ, MEIJI Techno Co. LTD, www.meijitechno.com) with ultraviolet light, and examined for the presence of colored fluorescent powder. Every bee was scored either positive or negative for the presence of colored powder. If a powder was detected on a bee, the color of the mark was recorded.

### Detection of protein powders

After each individual bee was visually examined for the presence of colored powder, it was placed into a 1.5 mL microcentrifuge tube containing 1000 µL of tris-buffered saline (TBS, pH 7.4) and soaked for ≥ 1 hour at 120 rpm on an orbital shaker. Each sample was then analyzed for the presence of egg albumin and milk casein by the protein-specific ELISAs described below (Jones et al. 2006).

### Anti-egg albumin ELISA.

A 100 µL aliquot of the solution that the bee was soaked in was placed in an individual well of a Falcon Pro-Bind™ 96-well ELISA plate (Becton Dickinson and Company, www.bd.com). Each ELISA plate was incubated for one hour at 37 °C. The contents of each well were discarded and washed 5× with a tris-buffer saline-tween 20 (TBST, 0.5% tween, pH 7.4) solution (Sigma-Aldrich, www.sigmaaldrich.com) Then, 360 µL of a TBS-bovine serum albumin (1.0% BSA, pH 7.4) (Sigma-Aldrich) solution was added to each well to block any remaining non-specific binding sites on the plates. Each plate was incubated for one hour at room temperature or overnight at 4 °C. The blocking solution was discarded, and each well was washed 2× with TBST. A 50 µL aliquot of rabbit anti-chicken egg albumin (ovalbumin) (Sigma-Aldrich) diluted 1:8000 in a buffer solution consisting of TBS-BSA (1%) and Silwet L-77 (Setre Chemical Company) (1.3 µL/mL) was added to each well for one hour at 37 °C. The antibody was discarded and the plates were again washed 5× as described above. A 50 µL aliquot of goat anti-rabbit IgG (whole molecule) (Sigma-Aldrich) conjugated to horseradish peroxidase diluted 1:2000 in the TBS-BSA-Silwet buffer described above was added to each well for two hours at 37 °C. The secondary antibody was discarded, the plates were washed 5× with TBST, and a 50 µL aliquot of TMB 1 Component HRP Microwell Substrate (SurModics, www.biofx.com) substrate was added to each well for 10 min at 27 °C. Following substrate incubation, the optical density of each well was measured with a SpectraMAX 250 microplate reader (Molecular Devices, www.moleculardevices.com) set at 650 nm.

**Anti-casein ELISA.** A 100 µL aliquot of each bee sample solution was placed in an individual well of a 96-well ELISA plate. The assay plate was incubated for one hour at 27 °C. The contents of each well were discarded and washed 2× with a TBST solution. 360 µL of a 25% chicken egg white solution diluted with TBS was then added to each well to block any remaining non-specific binding sites on the plates. Each plate was incubated for one hour at 4 °C. The blocking solution from each plate was discarded and washed 2× with TBST. A 50 µL aliquot of sheep antibovine casein (Meridian Life Science, www.meridianlifescience.com) diluted 1:2000 in a buffer solution consisting of 25% chicken egg white solution in the TBS solution was added to each well for one hour at 4 °C. The antibody was discarded, and the plates were washed 5× with TBST and a 50 µL aliquot of mouse anti-goat/sheep IgG (Sigma-Aldrich) conjugated to horseradish peroxidase diluted 1:4000 in a 25% egg white solution in the TBS buffer was added for one hour at 4 °C. The secondary antibody was discarded, plates were washed 5× with TBST, and a 50 µL aliquot of TMB substrate was added to each well for 10 min at 27° C. The optical density of each well was measured as described above.

### Honey bee negative controls

Honey bees serving as negative controls (n = 8 per ELISA plate) were collected from unmarked colonies located at the Carl Hayden Honey Bee Research Laboratory, Tucson, AZ, USA. Negative control bees were visually examined for the presence of fluorescent powder and then assayed for the presence of each protein mark by the ELISAs described above. Mean (±SD) ELISA optical density values were calculated. Individual honey bees collected at the study site were scored positive for protein if the ELISA optical density value was six standard deviations above that of the negative control mean.

### Data analysis

The efficacy of the various markers was determined by recording the percentage of fluorescent- and protein-marked bees (1) exiting unmarked hives, (2) exiting marked hives, and (3) flying in the vicinity of each apiary. Descriptive statistics are shown for the quantitative ELISA results yielded from only those bees collected within apiaries containing one or the other of the two protein marks (i.e., apiaries 6 through 9). Each bee was first scored either positive or negative by each ELISA for the presence of each respective mark. Then, the mean (±SEM) ELISA optical density values were graphed for bees that scored positive and negative to depict the difference between marked and unmarked bees.

A Chi-square (χ^2^) calculation with Yates' correction for continuity (Glantz 1997) was conducted to determine if the observed number of marked bees was significantly different than the expected number of marked bees at the entrance of unmarked hives, at the entrance of marked hives, and within the vicinity of each apiary. None of the bees collected at the entrances of unmarked hives were expected to contain a mark, while all the bees collected from the entrances of the marked hives were expected to contain a mark. The proportion of marked bees flying within the vicinity of each apiary was expected to be equal to the proportion of marked hives in each respective apiary (Table 1). Since the expected value for the number of marked bees obtained from the unmarked hives was zero, all data were transformed by adding 1 to both the expected outcome and the observed outcome of observations to eliminate the 0 from the denominator in the χ^2^ calculation. The data presented in the tables is the non-transformed values obtained from the experiment.

## Results

### Bee marking apparatus

**Figure 3.**
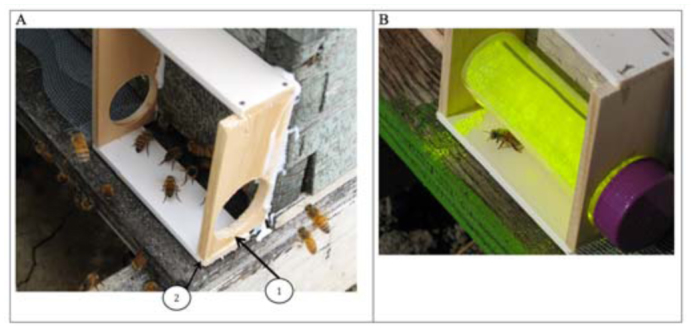
(A) A photograph of a bee marking device attached to the entrance of a honey bee colony without the powdered marker dispenser tube and (B) a photograph of a powdered marker dispenser tube containing Saturn Yellow fluorescent powder placed in a marking device. In photograph A, note the screen used to block the remaining length of the hive entrance and also note the gap between the hive entrance and the bottom piece of the marking device which forces the bees to step up and come in contact with the dispenser tube when it is in place. High quality figures are available online.

A photograph of a bee marking device (without a dispenser tube) attached to a beehive is shown in Figure 3A. There are two subtle, but critical, features of this design that make it effective for marking honey bees. First, the bottoms of the holes on the two vertical sides of the device were cut precisely 5.0 mm above the bottom edge of each lath (Figure 3A-1). Second, the bottom horizontal lath was attached to the front edge of the vertical laths (Figure 3A-2), leaving a gap toward the hive entrance. These two features were designed to facilitate transfer of the marker from the dispenser tube to the bees, because it forced the bees to step up onto the platform of the device and then squeeze under the sagging fabric mesh dispensing the powdered marker as they departed the hive. A photograph of a powder dispenser tube inserted into the marking device is shown in Figure 3B. A Video of honey bees exiting the hive through the marking device is shown here.

**Video 1.**
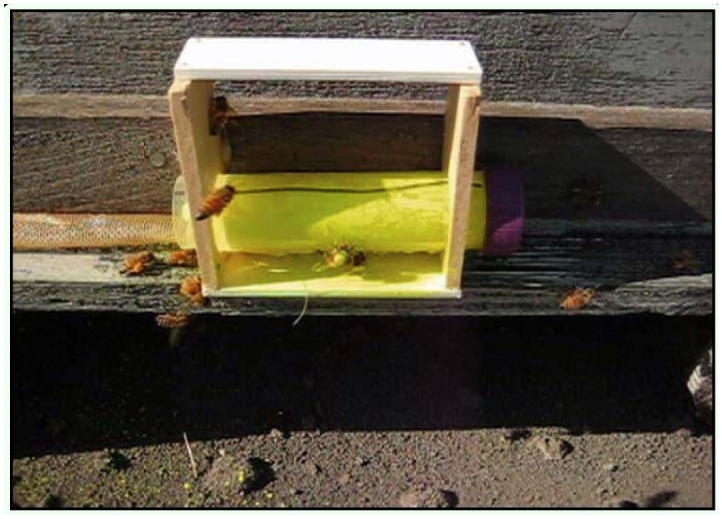
Click image to view video. Download Video

There are also two subtle features of the design of the dispenser tube that made it effective. First, the muslin cloth attached to the bottom of the dispenser had a fine enough mesh to hold the dust in the device until a bee rubbed against it (e.g., a fine sprinkle of dust was administered on top of the bees as they exited the hive). Second, the fabric glued onto the dispenser tube was cut slightly larger than the opening to create a pouch, or cushion-like effect. This ensured that bees had to squeeze between the bottom platform of the device and the marking powder dispenser tube as they exited the hive. Although the 50 mL dispenser tube contained enough powder to mark bees for several days, the powder had a tendency to clump within the tube under field conditions. Each day, prior to initiation of honey bee flight activity, tubes should be removed from the device, shaken to break up any clumps, and reinserted into the device.

### Analysis of bees exiting unmarked hives

A total of 139 bees were collected as they exited unmarked beehives from five of the nine apiaries. Every bee was examined, first visually for the presence of fluorescent colored powder and then immunologically by protein-specific ELISAs for the presence of both proteins. The bees exiting an unmarked hive should not be marked. Hence any bee collected from the entrance of an unmarked hive that contained a mark of any kind was regarded as “false positive” for the presence of a mark. Overall, 12 bees (8.6%) contained fluorescent powder and two bees (1.4%) contained one or the other type of protein (Table 2). Only the number of green and magenta marks found on bees collected from the unmarked hive entrances deviated from the expected outcome of zero. Specifically, 20.0 and 11.9% of these bees possessed a green (χ^2^ = 12.83, df 1, *p* < 0.01) or magenta mark (χ^2^ = 42.95, df 1, *p* < 0.01), respectively. The color detected on each bee exiting from an unmarked hive was the same as the color marker placed at the entrances of other nearby hives in each apiary (note that the marked hives were generally located 1 to 25 m from the unmarked hives). As such, these “false positive” reactions were inconsequential to the ultimate goal of our study, which was to mark as many bees as possible in each apiary with a distinctive mark. However, the two bees yielding a “false positive” immunoreaction represent true false positive reactions, because there were no hives nearby that contained a protein mark (Figure 2).

**Figure 4.**
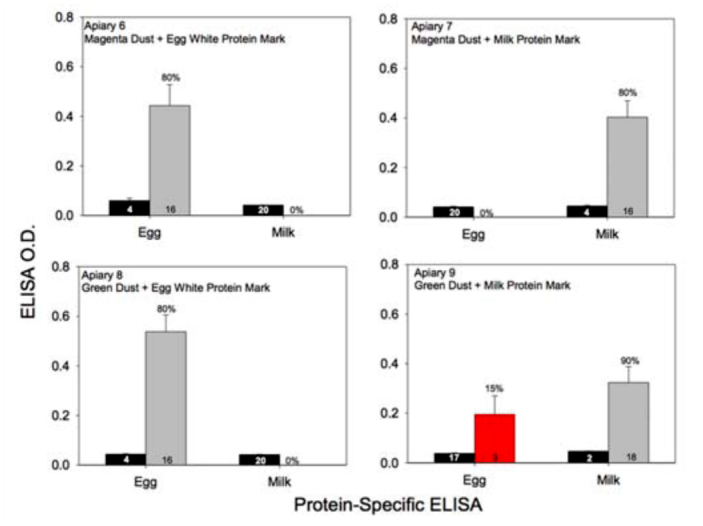
Mean (±SEM) ELISA optical density values for either chicken egg albumin (apiaries 6 and 8) or milk casein (apiaries 7 and 9) yielded from bees that were collected by hand at hive entrances marked with one or the other protein. The percentage of bees scoring positive is given above the error bars of each gray vertical bar. The number inside each vertical bar is the sample size for each treatment. The red vertical bar shown in apiary 9 represents the mean ELISA optical density value yielded by three false positive ELISA reactions (e.g., three bees that should have been marked with milk casein protein, but yielded a positive reaction for the presence of egg albumin protein). High quality figures are available online.

### Analysis of bees exiting marked hives

A total of 183 bees were collected as they exited marked beehives from each of the nine apiaries. Again, every bee was examined visually for colored powder and then immunologically for each type of protein. The bees collected at the entrance of a marked hive should be marked. Hence, any bee not containing the targeted mark was classified as “false negative.” As expected, almost every bee (98.9%) contained the targeted fluorescent powder mark and most (80–90%) of the bees collected from those apiaries that were also marked with protein containing the targeted protein mark (Table 3). However, in some instances the observed number of protein-marked bees was significantly different than the expected outcome. The color detected on each marked bee was the same as the marker color placed at the hive entrance, and there were only 2.7 and 1.1% false positive egg albumin and milk casein protein-marked bees, respectively (Table 3). The mean ELISA optical density values yielded by the bees collected at the protein-marked apiaries (apiaries 6 through 9) are given in Figure 4. The mean optical density values ranged from 0.54 ± 0.06 for egg whites at apiary 8 to 0.32 — 0.06 for milk at apiary 9. Those individuals scoring negative, regardless of the ELISA, consistently yielded optical density readings of ≤ 0.05 (Figure 4), which was the same average optical density readings yielded by the negative control bees (data not shown).

### Analysis of free flying bees

**Figure 5.**
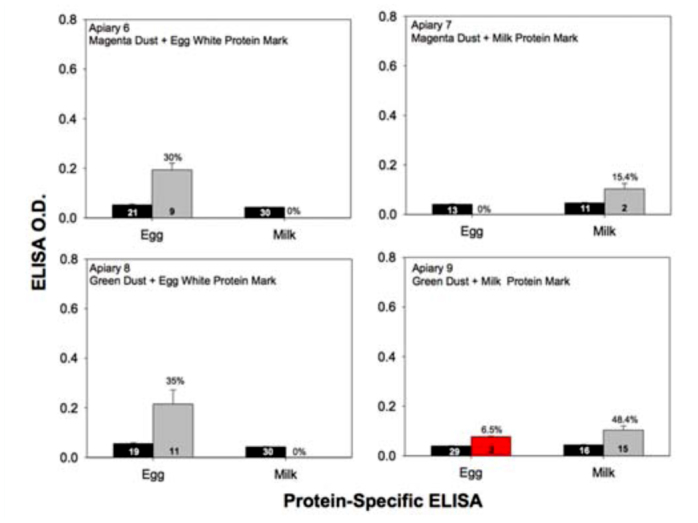
Mean (±SEM) ELISA optical density values for either chicken egg albumin (apiaries 6 and 8) or milk casein (apiaries 7 and 9) yielded from bees that were collected by sweep net while flying within the periphery of apiaries marked with one or the other protein. The percentage of bees scoring positive is given above the error bars of each gray vertical bar. The red vertical bar shown in apiary 9 represents the mean ELISA optical density value yielded by two false positive ELISA reactions (e.g., two bees that should have been marked with milk casein protein, but yielded a positive reaction for the presence of egg albumin protein). High quality figures are available online.

A total of 294 bees in flight collected within the vicinity of each of the nine apiaries were examined for the presence of any type of mark. These free flying bees were assumed to be the incoming and outgoing foragers at each apiary. Hence, the percentage of marked bees in flight was expected to approximately equal the percentage of marked hives at each apiary location (see Table 1). In apiaries 1 through 5, there was 100% fidelity of the fluorescent mark (e.g., every marked bee contained the targeted mark for that apiary), and very few false positive (1.7%) protein-marked bees. The observed percentage of free flying bees possessing a fluorescent colored mark was often significantly higher than the expected outcome in those apiaries. In apiaries 6 through 9, where all the hives were marked with green or magenta powder and an egg or milk protein powder, the observed percentage of green marked bees almost always met expectations, but the magenta marked bees did not. Also, the observed percentage of protein-marked bees always fell below the expected outcome of 100%. For example, only 30% (χ^2^ = 433.81, df 1, *p* < 0.01) and 36.7% (χ^2^ = 353.29, df 1, *p* < 0.01) of the individuals collected in the vicinity of apiaries 6 and 8 contained egg albumin, and only 15.4% (χ = 118.13, df 1, *p* < 0.01) and 48.4% (χ^2^ = 247.76, df 1, *p* < 0.01) of the bees collected in the vicinity of apiaries 7 and 9 contained casein (Table 4). Mean ELISA optical density values from samples of bees flying within the periphery of the protein-marked apiaries is given in Figure 5. The ELISA values for positively marked bees ranged from 0.22 ± 0.06 for egg whites at apiary 8, to 0.10 ± 0.02 for milk at apiary 9. Again, the free flying bees scoring negative by ELISA consistently yielded optical density values of < 0.05.

## Discussion

Simultaneously mass marking honey bees at different locations and then monitoring their dispersal is problematic. Many devices have been developed over the past half-century for marking bees (Smith and Townsend 1951; Dhaliwal and Sharma 1972; Howpage et al. 1998; Martin et al. 2006). Generally, these devices facilitated the self-marking of bees with a single type of mark as they exited the hive. Marking honey bees becomes more complicated if multiple marks are needed to distinguish among bees originating from many different locations spread over a vast area. In this study, we described the development of a portable honey bee marking device that can be rapidly deployed and used to reliably deliver a wide variety of powdered markers to bees as they exit the hive. The portability of the device was key to ensuring that the bees spread over the vast research area were marked as they initiated flight each day, i.e., the dispenser tubes could be rapidly loaded in devices previously attached to numerous hives before the onset of bee flight each day. Ultimately, this device was useful in enabling identification of the origin and distance traveled by field-collected bees originating from these nine different apiaries surrounding alfalfa seed production fields as depicted in Figure 2 (Hagler et al. 2011).

Variously colored fluorescent powders have been the most common markers used for bee mark-capture research (Southwood 1978; Hagler and Jackson 2001). Fluorescent powders are convenient, because they are easy to apply, easy to detect, and available in a wide variety of colors. They also have no negative impact on colony health or hive products. In a pilot test, many elaborately named DayGlo™ (DayGlo, www.dayglo.com) fluorescent markers placed on honey bees (e.g., Arc Yellow (which is actually orange), Blaze Orange, Corona Magenta, Saturn Yellow, Horizon Blue, Signal Green, Rocket Red, and Aurora Pink) were examined for efficacy. Of these, only five colors were found to be clearly distinguishable when present in small quantities on honey bees. Since more than five clearly distinguishable marks were needed for the field dispersal study (Hagler et al., 2011), the bees were double-marked at some apiary locations by mixing either magenta or green fluorescent powder with either egg white or milk protein powder. The net result was that nine distinct marks were identified for uniquely labeling bees at each apiary location.

This study and others (Smith and Townsend 1951; Boylan-Pett et al. 1991; Howpage et al. 1998; Martin et al. 2006) show that fluorescent powders are excellent markers for honey bees. The five colored powders were easily detected on bees by visual inspection, with the aid of a dissecting microscope and ultraviolet light. Moreover, there is little or no likelihood of obtaining a falsely marked bee. Obviously, the colored marks are easier (e.g., don't require an assay) and less expensive to detect than the protein marks. However, the protein-specific ELISAs are relatively simple, standardized for mass production (e.g., > 1000 samples per day), quantifiable, and only cost about $0.50 per sample (Fournier et al. 2008). Therefore, the detection of protein by ELISA is less tedious and faster (which ultimately is less costly) if thousands of samples must be processed (Hagler et al. 2009).

This is the first time to our knowledge that dry protein powders have been tested as insect markers. The bees were double-marked at four of the nine apiary locations with a 1:1 mixture of either magenta or green fluorescent colored powder and either egg white (egg albumin protein) or milk (casein protein) powder. The protein powders could be used exclusively for marking bees, though they were not as reliable as the fluorescent powders. While there was only a slight chance (generally < 1.0%) of obtaining a false positive protein-marked bee (i.e., a bee that should not have contained a protein mark), there was a relatively high occurrence of false negative marked bees (i.e., a bee that should have had a protein mark, but did not). This was especially true for bees flying in the vicinity of the apiaries. The reason for the relatively high frequency of “false negative” ELISA reactions is unknown. Perhaps protein powder does not, for whatever reason, adhere to bees as well as fluorescent powder. Other plausible explanations might include subtle assay procedures that can be modified. One area that deserves further investigation is to test the effect of honey bee sample preparation on ELISA sensitivity. For the present study, we soaked the bees at 120 rpm for ≥ one hour. In contrast, Jones et al. (2006) soaked the much smaller pear psylla *Cacopsylla pyricola* for only one to three minutes by gently submerging individuals in the buffer. It is conceivable that a large amount of non-target bee protein or protein acquired by a bee (e.g., pollen, nectar, plant debris, etc.) could have been extracted during sample preparation. If so, this could reduce the sensitivity of the indirect ELISAs by competitive binding of the non-marking proteins onto the limited number of protein binding sites available on an assay plate. This issue might be resolved with the development of protein-specific sandwich ELISAs (Hagler 1998). Conversely, we may have inadvertently applied too much protein to the bees. Although this seems counterintuitive, it is possible that too much target protein in a sample can produce a phenomenon known as steric inhibition (Crowther 2001). This decreases the sensitivity of an ELISA when the antibodies are not able to bind to the antigens, because the marker molecules are too closely packed for attachment of the antibodies. Thus, less target protein added to the marking dispenser or a greater dilution of the bee sample may result in better ELISA response in certain circumstances.

Aqueous protein sprays and protein impregnated foodstuffs have proven very effective for marking a wide variety of insects (Hagler et al. 1992, 2002; Hagler 1997; Blackmer et al. 2004; Peck and McQuate 2004; Buczkowski and Bennett 2006; Jones et al. 2006; Hagler et al. 2009; Horton et al. 2009; Baker et al. 2010; Hagler and Jones 2010) including honey bees (DeGrandi Hoffman and Hagler 2000). We are confident that the protein marking procedure can be improved with further testing. Perhaps the use of a liquefied protein delivery system (Hagler and Jackson 1998; Hagler and Naranjo 2004; Jones et al. 2006; Hagler et al. 2009; Hagler and Jones 2010), a protein-baited food source (e.g., sugar syrup containing protein) (DeGrandi-Hoffman and Hagler 2000; Hagler et al. 2002, 2009; Baker et al. 2010), a different concentration, a different type of protein mark, or a different ELISA format (e.g., sandwich ELISA) would prove even more effective for marking bees (Hagler 1998). These are areas for further research.

In summary, the compact bee marking device described in this paper is inexpensive, easy to construct, and easy to install. The portable marker dispenser tube can be loaded with variously colored fluorescent or protein powders and inserted into and removed from the device in seconds. These features provide a means to mass mark bees with a multitude of different markers and facilitate the synchronous application of marks to dozens of honey bee colonies spread over a large area. Ultimately, this methodology will be used to study the spatial distribution of honey bees over a large commercial alfalfa seed production area, containing both genetically modified and non-genetically modified alfalfa fields.

**Table 1.**
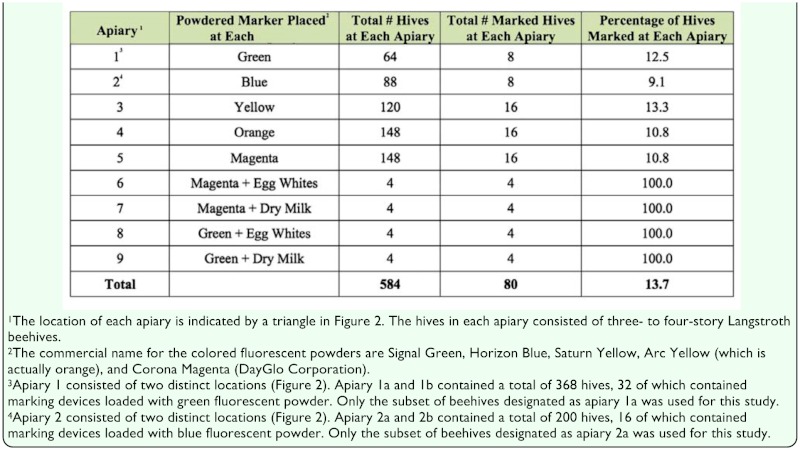
The total number of beehives, marked beehives, and the percentage of marked beehives located at each apiary.

**Table 2.**
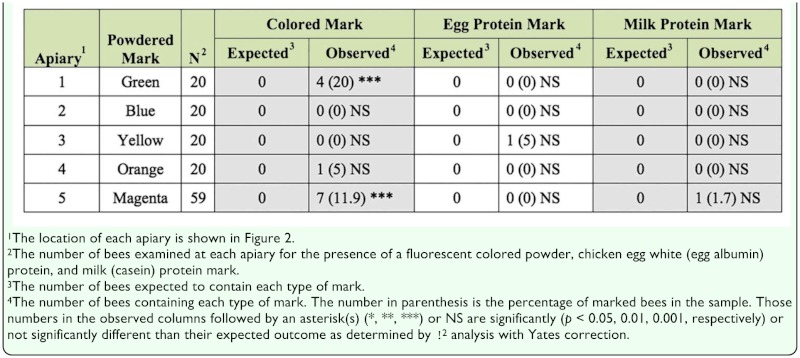
The expected and observed number of marked bees recovered from the entrances of unmarked beehives. Each individual bee was examined visually under magnification using ultraviolet light to detect the presence of a fluorescent colored powder mark, and then by an egg albumin and milk casein-specific ELISA to detect the presence of each type of protein mark.

**Table 3.**
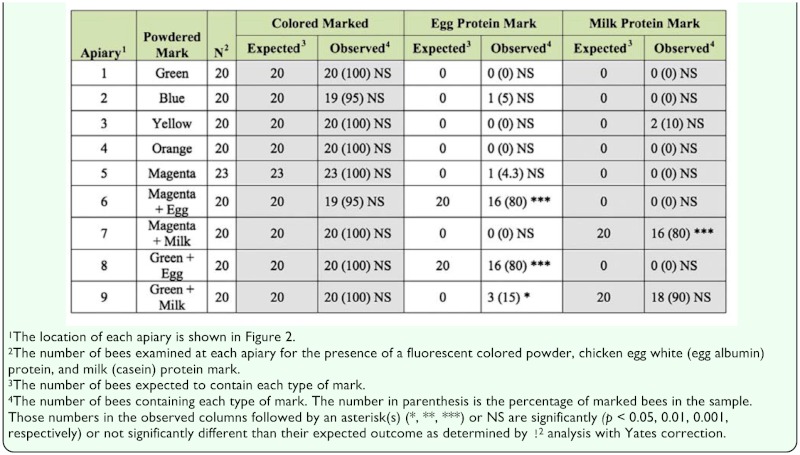
The expected and observed number of marked bees recovered from the entrances of marked beehives. Each individual bee was examined visually under magnification using ultraviolet light to detect the presence of a fluorescent colored powder mark, and then by an egg albumin and milk casein-specific ELISA to detect the presence of each type of protein mark.

**Table 4.**
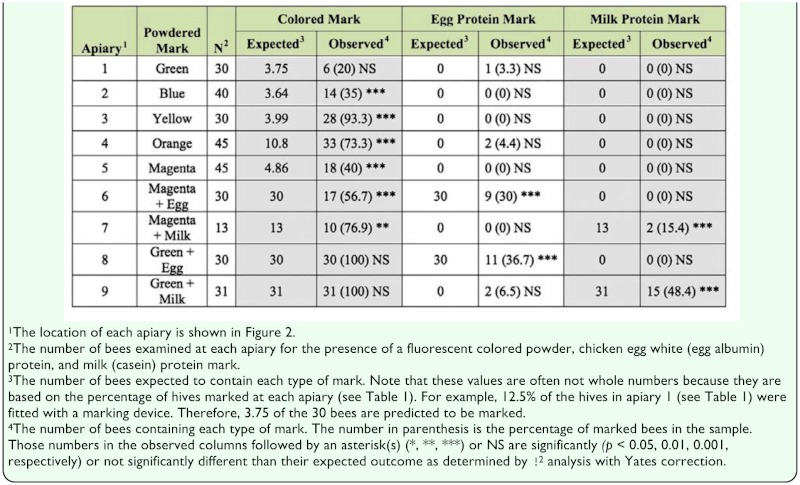
The expected and observed number of marked bees collected in flight within the periphery of each apiary. Each individual bee was examined visually under magnification using ultraviolet light to detect the presence of a fluorescent colored powder mark, and then by an egg albumin and milk casein specific-ELISA to detect the presence of each type of protein mark.
